# Treatment of Villous Adenoma With Underlying Adenocarcinoma of the Prostatic Urethra Using Combined Chemoradiation: A Case Report

**DOI:** 10.7759/cureus.64841

**Published:** 2024-07-18

**Authors:** Anthony J Corsi, Thomas P Bradley, Saroja Devi Geetha

**Affiliations:** 1 Internal Medicine, Zucker School of Medicine, North Shore University Hospital/Long Island Jewish Medical Center, Northwell Health, Greenvale, USA; 2 Hematology and Oncology, Zucker School of Medicine, North Shore University Hospital/Long Island Jewish Medical Center, Northwell Health, Greenvale, USA; 3 Pathology, Zucker School of Medicine, North Shore University Hospital/Long Island Jewish Medical Center, Northwell Health, Greenvale, USA

**Keywords:** chemoradiation, prostate cancer, prostatic adenocarcinoma, prostatic adenoma, adenocarcinoma of prostate, adenoma of prostate, prostatic urethra, villous adenoma

## Abstract

The presence of villous adenoma in the urinary tract is an exceedingly rare finding. On a histological and cytological level, this tissue is essentially identical to that typically found in the colon. These lesions do have malignancy potential and, when present with coexistent adenocarcinoma, have a risk of recurrence and metastasis even after surgical resection. Although villous adenomas of the urinary tract have been almost exclusively treated with surgical intervention in the literature, we present a case of villous adenoma with underlying adenocarcinoma of the prostatic urethra that was successfully treated with combined chemoradiation therapy. While surgical excision has been shown to be curative in diseases with isolated villous adenoma, more aggressive treatment with radiation and/or chemotherapy can be considered in patients with concurrent adenocarcinoma. However, more research into this subject is required to properly determine the best choice of therapy for this niche patient population.

## Introduction

A villous adenoma is a benign tumor of glandular origin, typically found in the colon. It is a sessile tumor with protruding villi made up of stroma that is encased by a surface of columnar epithelial cells [1]. Approximately 70% of polyps removed from the colon are adenomas, 5% of which can further be classified as having villous morphology. Furthermore, the amount of villous component present is directly correlated with malignancy potential [2]. Although adenomas are frequently seen in the sigmoid colon, they may also rarely appear in the urinary tract, and villous adenomas have been documented in the urinary bladder, renal pelvis, ureter, and urethra. Typically seen in men older than 50 years old, these patients usually present with urinary symptoms such as changes in voiding behavior, hematuria, and mucosuria [2,3]. Treatment of these isolated cases has largely included either local transurethral resection or radical surgery, but the utilization of combined chemoradiation therapy has been rarely discussed [3,4]. In this case report, we describe the case of a 77-year-old male found to have evidence of a villous adenoma with underlying adenocarcinoma in the prostatic urethra that was successfully treated with radiation therapy and oral capecitabine.

## Case presentation

**Figure 1:**
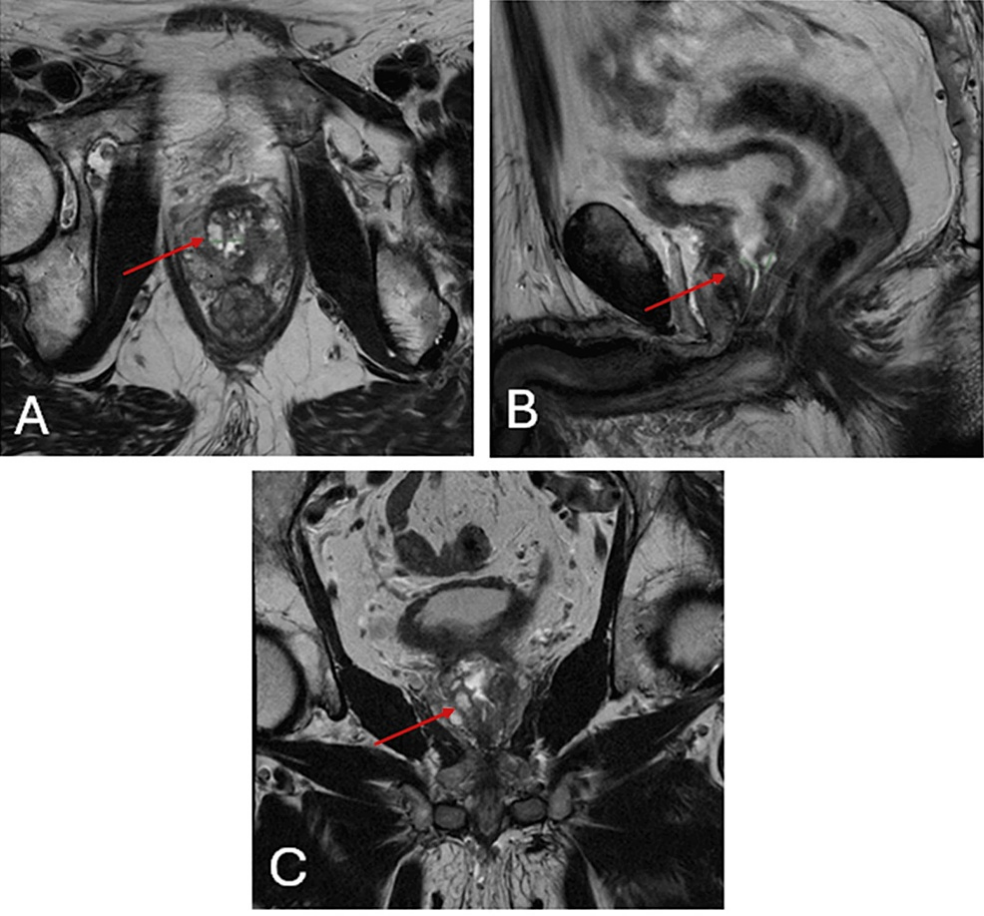
Axial (A), sagittal (B), and coronal (C) views of the MRI pelvis demonstrate enlargement of the prostate (red arrows), measuring 4.2 x 3.2 x 3.9 cm. MRI: magnetic resonance imaging

A 77-year-old male initially presented to his primary care provider for increased urinary frequency and urgency, mucosuria, and suprapubic pain. Subsequent workups revealed microhematuria, and a CT scan revealed findings consistent with benign prostatic hyperplasia and chronic cystitis, along with chronic bladder outlet obstruction. Cystoscopy further demonstrated a large bladder diverticulum with an intravesical median lobe and evidence of benign prostatic hypertrophy. Transurethral resection (TURP) and aquablation of the prostate were then performed, and a biopsy of the verumontanum and proximal prostate sinus revealed fragments of villous adenoma. A magnetic resonance imaging (MRI) of the pelvis showed a 4.2 x 3.2 x 3.9 cm prostate with a TURP defect and diffuse inflammatory-type enhancement throughout the peripheral zone (Figure 1).

**Figure 2:**
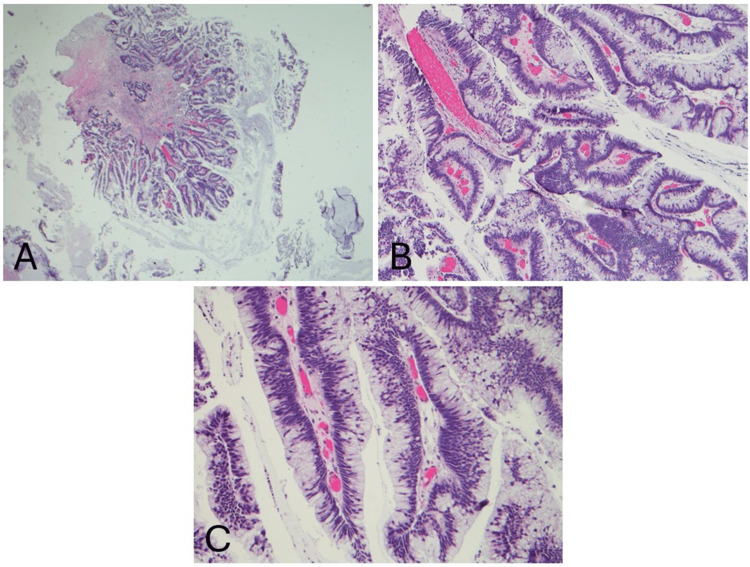
H&E staining reveals a villous adenoma with a background of extravasated mucin at 2× magnification (A), 10× magnification (B), and 20× magnification (C). H&E: hematoxylin and eosin

**Figure 3:**
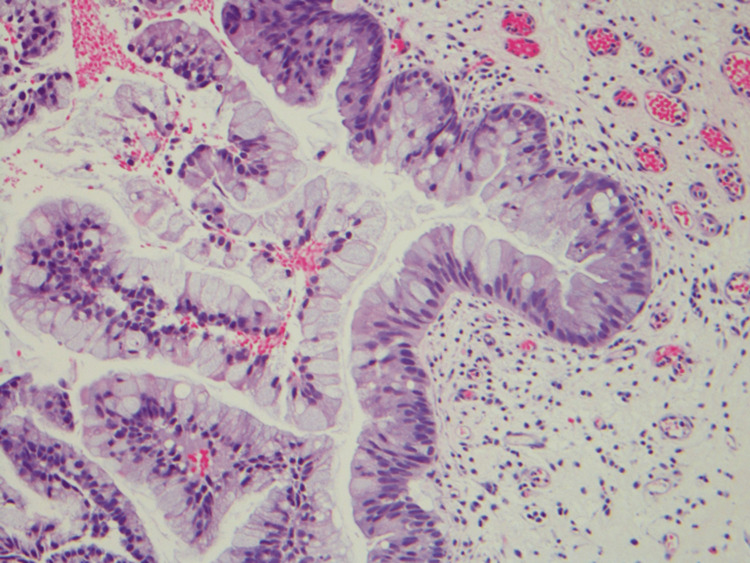
Mucinous epithelium with goblet cells at 20× magnification (H&E staining). H&E: hematoxylin and eosin

**Figure 4:**
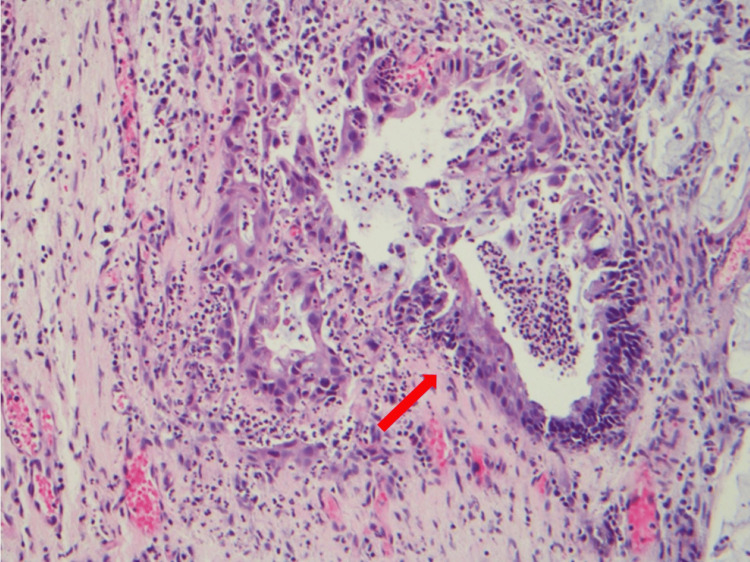
A malignant gland within the stroma (red arrow) representing adenocarcinoma at 20× magnification (H&E staining). H&E: hematoxylin and eosin

The patient then underwent a colonoscopy to rule out primary colorectal cancer, but this revealed only a small tubular adenoma without evidence of adenocarcinoma. He ultimately underwent a repeat cystoscopy with a biopsy of the prostatic urethra, again showing villous adenoma tissue, now with a focus of mucinous adenocarcinoma, moderately differentiated, without lymphovascular invasion (Figures 2, 3, 4).

**Figure 5:**
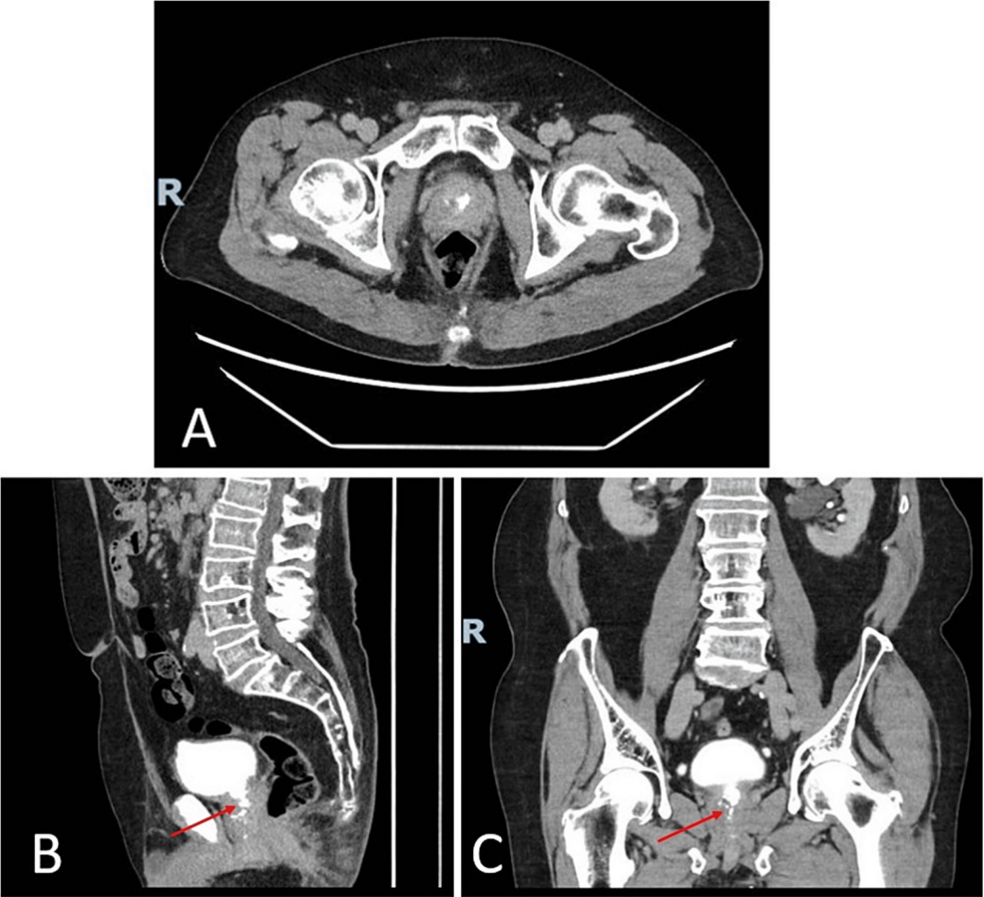
Axial (A), sagittal (B), and coronal (C) views of the CT of the chest, abdomen, and pelvis with urogram demonstrating a TURP defect of the prostate (red arrows) prior to starting combined chemoradiation.

A CT of the chest, abdomen, and pelvis with a urogram showed only a TURP defect of the prostate without evidence of metastatic disease (Figure 5). The patient declined radical prostatectomy at that time and was instead referred to medical and radiation oncology for further management. Combined chemoradiation therapy was subsequently initiated with the patient receiving 5000 cGy in 25 fractions to the pelvis and regional urethral tissues, along with capecitabine 1,500 mg twice daily, five consecutive days a week, over five weeks. Following the completion of treatment, a repeat cystoscopy with cytology performed at 2.5-, 6.5-, 9-, and 12-month intervals showed no evidence of recurrent disease. Moreover, the CT of the chest, abdomen, and pelvis with urogram completed 4.5 and 11 months post-treatment were negative for signs of metastatic disease.

## Discussion

The presence of villous adenoma in the urinary tract is a rare finding. Histologically, villous adenomas appearing in the urinary tract are identical to those typically seen in the colon. The morphology includes similar finger-like villi and fibrovascular stroma, and the nuclei of the epithelial cells observed also exhibit comparable hyperchromasia and loss of nuclear polarity [3,4]. The pathogenesis of these tumors is still not fully known, but multiple theories exist. One suggests that the link is derived from the shared embryological origins of the bladder and rectum. During embryogenesis, the cloaca of the endoderm is divided by the urorectal septum into what becomes the bladder and urinary tract ventrally and the rectum dorsally. While the former differentiates into a transitional epithelium and the latter into columnar cells, the presence of a cloacal remnant retained in the urinary tract can theoretically expand into a glandular neoplasm [5,6]. Alternatively, it is also proposed that these tumors are the result of the typical metaplasia, dysplasia, and then carcinoma transition sequence that occurs in the setting of chronic irritation. Among some cases of villous adenoma in the bladder, there was evidence of surrounding cystitis glandularis, a finding of chronic inflammation that occurs with persistent tissue irritation. This was further supported by the presence of neutral mucins, acidic sulfomucins, and sialomucins found in both the villous adenoma and adjacent areas of cystitis glandularis [3,7]. In a case of chronic cystitis showing evidence of intestinal-type glandular metaplasia and some areas of dysplasia, a tubulovillous adenoma was found as well.

Flow cytometry further demonstrated similarly increased expression of certain DNA markers, such as proliferating cell nuclear antigen and MIB-1, in both the villous adenoma and the dysplastic areas within the metaplastic mucosa, suggesting that the adenoma tissue may have originated in the background of intestinal metaplasia [8]. While these studies support the theory of progression from metaplasia to dysplasia, long-term follow-up studies of intestinal metaplasia have yielded mixed results. While intestinal metaplasia found with coexistent dysplasia has the potential risk of progression to carcinoma, findings of metaplasia alone have not been shown to have the risk of further disease advancement [9,10].

Villous adenomas of the urinary tract have almost always been treated with surgical intervention in the literature [3,5,11-20]. The use of chemotherapy and radiation has been documented once, but the details of the therapy regimen, including the chemotherapy agent used, the dose of radiation, and the scheduling of each, were not mentioned. Furthermore, this single patient suffered multiple disease recurrences and passed away from unknown causes approximately six years after the initial diagnosis [3].

Here, we report a case outlining the treatment of a villous adenoma with features of mucinous adenocarcinoma that did not result in the recurrence of the disease thus far at one year post-treatment. When a villous adenoma is found to have concurrent adenocarcinoma, it has been shown that there is an increased risk for disease recurrence or distant metastasis even after surgical intervention [3,5]. This may then suggest that more aggressive therapy may be warranted when villous adenoma tissue is found with coexistent adenocarcinoma, such as in our case. Although not discussed in the literature, radiation and/or chemotherapy can then be considered in patients found to have coexistent adenocarcinoma or simply in those who decline radical surgical intervention. Since these tumors are seemingly identical to those found in the colon, the use of fluoropyrimidine agents capecitabine or 5-fluorouracil may be appropriate. However, further research is required to discern both the best choice of chemotherapy agent and, more importantly, how long-term outcomes differ between those who undergo surgical intervention and those who receive additional adjuvant radiation and/or chemotherapy.

## Conclusions

A villous adenoma of the urinary tract is a rare entity indistinguishable from its colonic counterpart that conveys malignancy potential. In patients with coexistent adenocarcinoma or for those who decline radical surgery, treatment with chemoradiation may be a possible alternative to surgery for curative therapy. Although we present a case of successful treatment of villous adenoma with underlying adenocarcinoma of the prostatic urethra, further research into the choice of therapy regimen and long-term outcomes is required for definitive recommendations in future clinical practice.
